# Synthesis and characterization of novel solid-supported salicylate-based ionic liquid for adsorptive removal of Pb(ii) and Ni(ii) ions from aqueous solution

**DOI:** 10.1039/d3ra00806a

**Published:** 2023-04-11

**Authors:** Nur Anis Liyana Kamaruddin, Mohd Faisal Taha, Avisenna Muhammad Romil, Fairuz Liyana Mohd Rasdi

**Affiliations:** a Centre of Research in Ionic Liquids, Universiti Teknologi PETRONAS Seri Iskandar Perak Malaysia; b Fundamental and Applied Sciences Department, Universiti Teknologi PETRONAS Seri Iskandar Perak Malaysia

## Abstract

Novel solid-supported ionic liquid (Si-Sal-SSIL) was synthesized by immobilization of 1-methyl-3-(3-trimethoxysilylpropylimidazolium) salicylate [MTMSPI][Sal] ionic liquid onto the activated silica gel. First, the [MTMSPI][Sal] ionic liquid was derived from the reaction of a metathesis product of 1-methyl-3-(3-trimethoxysilylpropylimidazolium) chloride [MTMSPI][Cl] with sodium salicylate through an ion-exchanged reaction. [MTMSPI][Sal] was purified and characterized through ion-chromatography, CHN and Karl-Fischer titration analyses. Further characterizations on [MTMSPI][Sal] were carried out by ^1^H NMR and FTIR analyses. Si-Sal-SSIL was successfully prepared and confirmed through BET and solid-state NMR analyses. Si-Sal-SSIL showed better removal capacities towards Pb(ii) and Ni(ii) ions in comparison to native activated silica gel. Si-Sal-SSIL was then applied as solid adsorbent for an efficient removal of Pb(ii) and Ni(ii) from the aqueous solution. A series of batch sorption study were performed to explore the influence of parameters *i.e.*, loading ratio of activated silica gel to [MTMSPI][Sal], pH, mixing time, initial concentration of analyte towards the adsorption of Pb(ii) and Ni(ii) ions onto Si-Sal-SSIL as a function of removal efficiency. Under optimized conditions, the sorption kinetics for removal of both metals agreed with pseudo-second order linear plots. The mechanism of Pb(ii) and Ni(ii) sorption by Si-Sal-SSIL gave good fits for Langmuir model.

## Abstract 2

Novel solid-supported ionic liquid (Si-Sal-SSIL) was synthesized by immobilization of 1-methyl-3-(3-trimethoxysilylpropylimidazolium) salicylate [MTMSPI][Sal] ionic liquid onto the activated silica gel.

## Graphical abstract

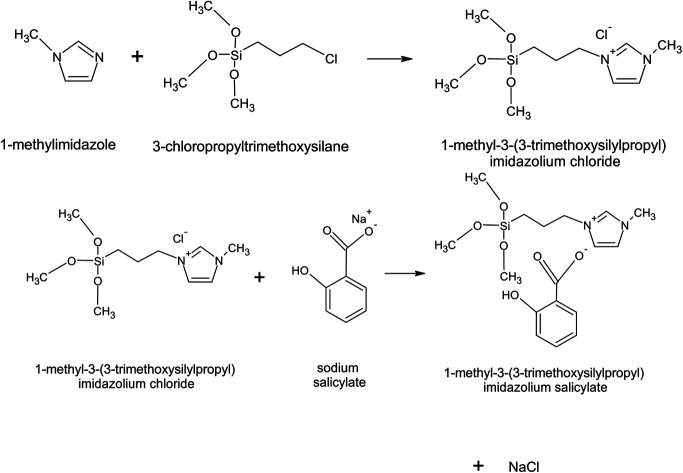

## 1 Introduction

Heavy metal concentrations in surface water, groundwater, and seawater have risen in tandem with unplanned urbanization,^1^ dumping of untreated waste/municipal waste,^2^ agricultural activity^3^ and other industrial processes^4^ such as mining, metal/steel production, and electroplating. Every year, industrial activity releases metric tonnes of heavy metals, which end up in water supplies all around the world. The emergence of excessive amounts of these non-degradable and persistence substances in water resources are a worldwide public health concern since those pollutants can be accumulated notably through food chain giving adverse effects to human health.^5^ Having an atomic number greater than 20 and a density greater than 5 g cm^−3^, heavy metals are a class of metals and metalloids that are toxic even at parts per billion (ppb) level. Examples include arsenic (As), cadmium (Cd), chromium (Cr), copper (Cu), iron (Fe), manganese (Mn), mercury (Hg), nickel (Ni), lead (Pb), zinc (Zn) and silver (Ag).

Nickel (Ni) metal production is expanding due to the increased usage of nickel (Ni) metal in the production of superalloys, lithium-ion, and nickel-metal hydride batteries for electric vehicles. Ni is utilised in approximately 8% of all household appliances, in addition to being employed in some pigmentation industry applications.^6^ Essentially, the manufacturing of Ni has resulted in the formation of a substantial amount of liquid/solid waste (*i.e.*, slag and leaching residues).^7^ Lead (Pb) is yet another heavy metal that is widely employed in a number of industries such as electroplating, painting, steel, batteries, smelting, inorganic fertilisers, and pesticides.^8,9^ The use of Pb in industrial operations, similar to the usage of Ni, has resulted in a significant volume of Pb tainted effluent that could leach into land areas and water bodies.^10^ Inadequate disposal of Ni and Pb effluent/wastewater may have devastating effects on environmental and human health. The permissible limit for Ni and Pb set by WHO (2012) for drinking water were only 50 μg L^−1^ and 10 μg L^−1^; respectively.^6^ Both metals are able to accumulate in the tissues of living organisms, and they had a high propensity to cause damage to a variety of tissues, including the kidney, the liver, and the brain.^11^ In severe cases, this damage could result in death.

Generally, several techniques have been applied for the removal of heavy metals in aqueous solution for example adsorption, reverse osmosis, reduction, chemical precipitation, ultrafiltration, flocculation, electrochemical treatment, solvent extraction, and ion exchange.^12,13^ However, these techniques suffered from few drawbacks such as high cost, lower efficiency, and require tedious operating conditions.^14–16^ Adsorption using suitable adsorbent has become a promising option for the removal of metal ions from aqueous solution/water/wastewater source due to its advantageous: availability, cost efficient, flexible in design of adsorbent, ease of operation and does not generate secondary pollutant.^17–19^ The mesoporous silica nanoparticles (MSNs) have drawn much attention as promising adsorbent with a few unique properties such as large surface area, good chemical stabilities, tuneable pore diameters, ease of surface modification, economical regeneration, and reusability.^20–22^ However, due to its lack of selectiveness, the surface modified MSNs technology has been developed. This approach has been shown to overcome the low loading capacity and weak interaction with metallic cation, as demonstrated by the low metal ion binding constant, resulting in a highly effective sorbent for heavy metal ion removal.^23^

In recent years, there has been an increase in the utilization of new class of metal extractant from aqueous solutions called ionic liquids (IL).^24,25^ ILs are molten salts with melting temperature lower than 100 °C. It consisted of two parts which are cation (bulky and non-symmetrical organic cations) and anion (organic/inorganic anions).^26^ ILs are considered as environmental benign solvents for its high thermal stability, low flammability, negligible vapor pressure and good dissolution properties.^27^ Nonetheless, large-scale use of ILs as metal extractants is challenging due to their viscosity, high cost, high consumption, and poor recovery.^28^ In addition, ionic liquids may leach into the aqueous phase, which increases cost due to loss of solvent and may pose an environmental risk.^29^ The high viscosity of ionic liquids causes lower extraction efficiencies due to the difficulty of diffusion of metal ions and low interaction with the aqueous phase.^30^ To overcome the drawbacks, ILs have been immobilized onto silica/polymeric supports in order to leverage their chemical functionality and successfully employed in different field of applications.^31^ From the perspective of large-scale industrial operations, solid supported ionic liquid (SSIL) can be easily separated from wastewater through filtration. Besides high surface area, studies have shown that SSIL has ideal thermal and mechanical stability characteristics.^32^

In this study, the salicylate-based ionic liquid, 1-methyl-3-(3-trimethoxysilylpropylimidazolium) salicylate ([MTMSPI][Sal]), was synthesized through a metathesis reaction. After [MTMSPI][Sal] was confirmed to be obtained with good purity, [MTMSPI][Sal] was chemically immobilised on activated silica to form a novel solid-supported ionic liquid (SSIL). In this piece of work, [MTMSPI][Sal], an IL containing salicylate functional group was chosen to be chemically immobilised in SSIL as an extraction agent for Pb(ii) and Ni(ii) ions from aqueous solution due to the high affinity of salicylate functional group with its additional OH moiety towards metal ions.^33,34^ Meanwhile, Pb(ii) and Ni(ii) ions have been selected as these metal ions are commonly found in industrial wastewater. The adsorption kinetics for the removal of Pb(ii) and Ni(ii) ions from single aqueous solution were carried out to determine the adsorption isotherms.

## 2 Experimental

### 2.1 Materials and instruments

Silica gel, hydrochloric acid fuming 37%, diethyl ether, methanol, toluene, dichloromethane and acetonitrile were acquired from Merck, New Jersey, United States. Meanwhile, 1-methylimidazole, 3-chloropropyltrimethoxysilane (CPTMS), sodium salicylate (97% purity), Ni(ii) nitrate salt (97% purity) and Pb(ii) nitrate salt (99.99% purity) were purchased from Sigma-Aldrich, United States. The stock solutions of 200 mg L^−1^ were prepared individually for Pb(ii) and Ni(ii) aqueous solution. Eventually, further dilutions were prepared by diluting the stock solutions with distilled water. Concentration of Pb(ii) and Ni(ii) ions in aqueous solution were measured using atomic absorption spectrometer (AAS) from Agilent (Model 240 FS, Agilent Technologies, United States).

### 2.2 Synthesis of [MTMSPI][Sal] ionic liquid

The precursor of the desired final IL, [MTMSPI][Sal] is [MTMSPI][Cl]. To synthesise this, 4.13 g (25 mmol) of 1-methylimidazole was added to a round bottom flask. This flask was then be put into silicone oil bath at 80 °C. From there, 1 molar equivalent of 3-chloropropyltrimethoxysilane was added to the flask dropwise with stirring on. This mixture was then refluxed for 72 hours. To purify this chloride IL and get rid of any unreacted reactants or potential side products, diethyl ether was added to the flask and shaken. This mixture was transferred to a separation funnel and allowed to settle. The lower layer containing the IL was then extracted. This washing step was repeated another 2 times for a total of 3 washing steps. The washed product was then put into a rotary evaporator to remove any residual diethyl ether.

**Fig. 1:**
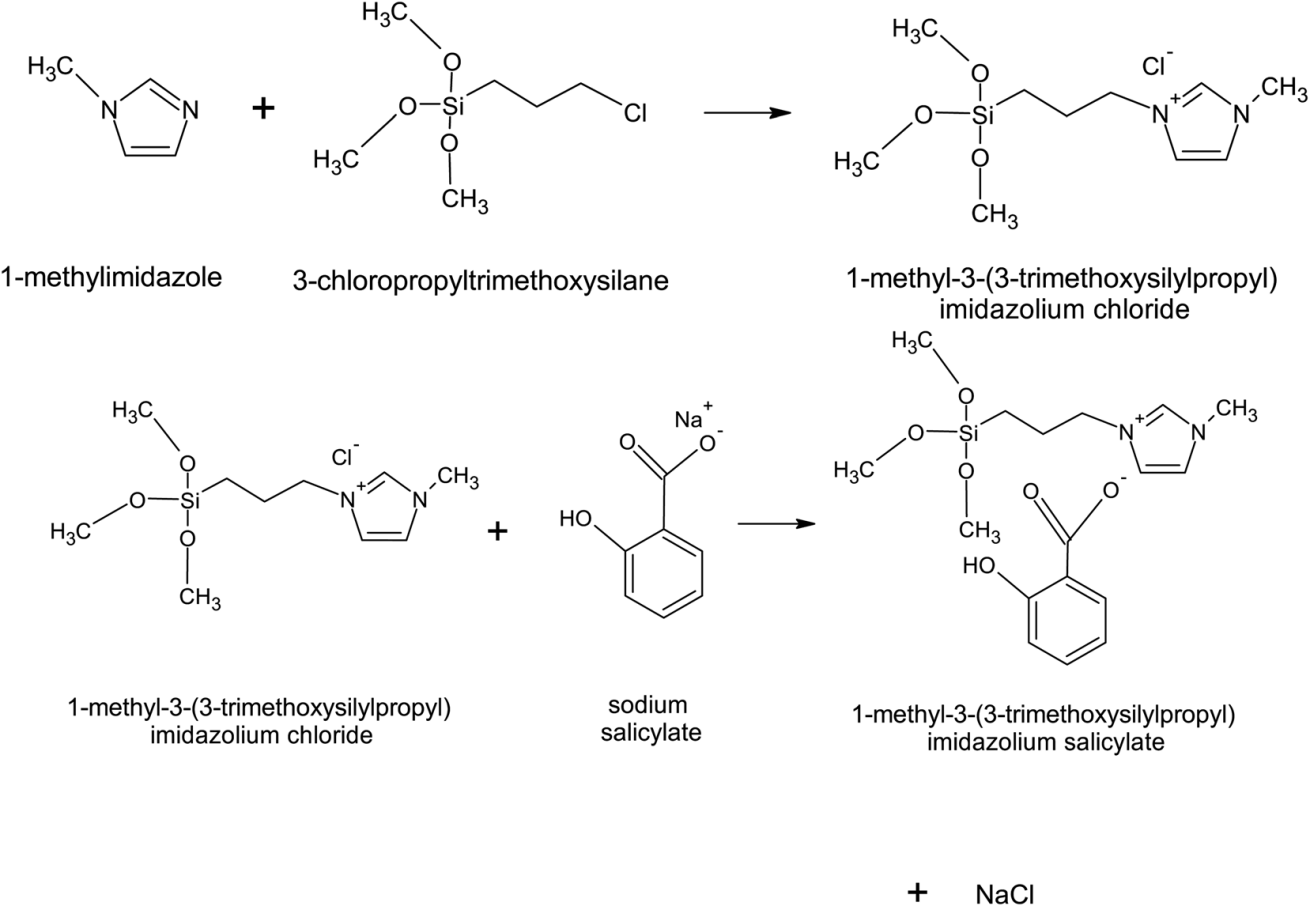
The synthesis route for the formation of newly synthesized [MTMSPI][Sal] ionic liquid through metathesis reaction.

A metathesis reaction was carried out to replace the chloride anion with the desired salicylate anion as shown in Fig. 1. For this, 25 mmol sodium salicylate was dissolved in water first before added to [MTMSPI][Cl] in a round bottom flask. This mixture was then stirred at 500–600 rpm for 48 hours to allow for complete reaction. To remove water content from the [MTMSPI][Sal], the product was dried in a rotary evaporator. Thereafter, acetonitrile was added to precipitate out the NaCl by-product. The precipitated NaCl was then filtered out using vacuum filtration. The filtrate still contained acetonitrile, and as such was dried using a rotary evaporator.

### 2.3 Immobilization of [MTMSPI][Sal] ionic liquid onto activated silica gel

To allow the [MTMSPI][Sal] ionic liquid to bind to the silica support material, the silica gel must be activated with strong acid to prepare activated silica gel. This was done by adding 10 g of silica gel to 100 mL of 6 M HCl in a flask and refluxing at 80 °C while stirring at 400 rpm for 8 hours. The mixture was then filtered through vacuum filtration to separate the activated silica gel from the mixture. To remove all acid from the prepared activated silica gel, the solid was washed 3 times with water, followed by 2 washings with ethanol. The washed solid was then dried in a vacuum oven at 70 °C and 500 mbar for 3 hours.

**Fig. 2:**
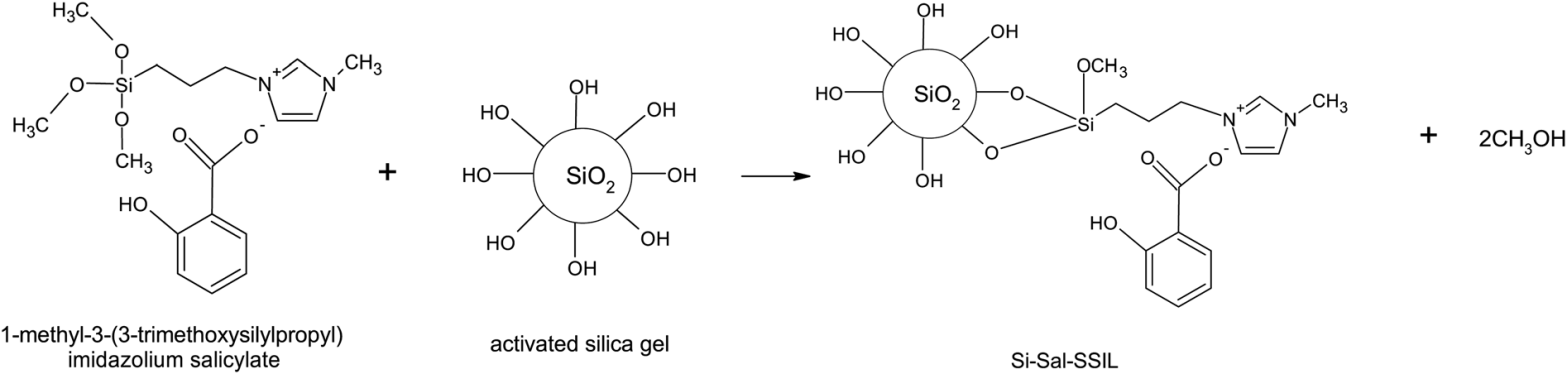
Synthesis route to produce Si-Sal-SSIL as the extractant to remove Pb(ii) and Ni(ii) ions from aqueous solution.

To immobilize the [MTMSPI][Sal] onto the activated silica gel to prepare the desired Si-Sal-SSIL extractant (Fig. 2), 5 g of the [MTMSPI][Sal] ionic liquid was first dissolved in a mixture of 20 mL of methanol and 20 mL of toluene. 10 g of activated silica gel was then added into the reaction vessel to obtain a loading ratio of silica gel to ionic liquid of 1 : 0.5. This mixture was then refluxed at 100 °C for 48 hours with stirring on to obtain a homogeneous mixture. The product was vacuum filtered and subsequently washed with dichloromethane to wash off any residual solvent and [MTMSPI][Sal] that did not chemically bind to the silica support material. Afterwards, the product, *i.e.*, Si-Sal-SSIL was placed in a vacuum oven at 120 °C overnight to dry. This procedure was repeated using different loading ratios of silica gel to ionic liquid (1 : 0.2, 1 : 0.12 and 1 : 0.1) to prepare Si-Sal-SSIL extractant for further study.

### 2.4 Characterization of [MTMSPI][Sal] ionic liquid and Si-Sal-SSIL extractant

The proton NMR spectra of novel [MTMSPI][Sal] ionic liquid was obtained in d-DMSO solvents through nuclear magnetic resonance (NMR) instrument (Model 1200 Series Avance III, Bruker, United States). Fourier-transform infrared spectrophotometer (FTIR) instrument (Model Frontier, Thermo Fisher Scientific, United States) was used to identify functional groups present in both [MTMSPI][Sal] ionic liquid and Si-Sal-SSIL extractant. The infrared spectra of the samples in potassium bromide pellets were recorded by accumulating 40 scans in the range of 4000 to 500 cm^−1^ with a resolution of 4 cm^−1^. The differences in carbon, hydrogen and nitrogen contents in [MTMSPI][Sal] ionic liquid was investigated using the combustible elemental analysis (CHNS Analyzer) (Model VarioMicro, Elementar, Hesse, Germany).

Furthermore, ion chromatography (IC) (Model Compact 930, Metrohm, Herisau, Switzerland) was utilized to determine the halide content in [MTMSPI][Sal], as halide, specifically chloride, is one of the ions from by-products. The purity of synthesized IL was ascertained with supporting Karl Fischer titrator (Model KF V30, Mettler Toledo, Columbus, OH, USA), which determines the moisture content in [MTMSPI][Sal] by coulometric method. Moreover, thermogravimetric analysis (TGA) (Model STA 6000, PerkinElmer, Waltham, MA, USA) was conducted to determine the thermal stability of the novel ionic liquid at a heating rate of 10 °C min^−1^ in a nitrogen atmosphere at temperatures ranging from 50 °C to 800 °C.

Apart from that, solid-state NMR analyses was employed to validate the presence of chemical bond between immobilized [MTMSPI][Sal] ionic liquid and activated silica gel. A 7 mm zirconium oxide rotor was used to compress the solid sample, which was then analysed using the magic angle spinning (MAS) technique at a frequency of 59.61 MHz for silicon. The cross-polarization (CP) technique was also utilized to improve the signal-to-noise ratio. For the acquisition of the ^29^Si NMR spectra, pulse repetitions of 3 seconds and contact times of 3 milliseconds were employed. Next, Brunauer–Emmett–Teller (BET) analysis (Tristar 3020, Micrometritics, Norcross, GA, USA) was performed to analyze the surface area of three samples that have different conditions each, namely, activated silica gel, Si-Sal-SSIL before washing with solvent, and Si-Sal-SSIL after washing with solvent, to confirm the elimination of physically immobilized [MTMSPI][Sal] from Si-Sal-SSIL extractant after solvent washing. The degassing temperature and holding time for all the samples were 150 °C and 300 min, respectively.

### 2.5 Batch adsorption study

A control study was first performed to compare the removal efficiency of three extractants *i.e.*, activated silica gel, [MTMSPI][Sal] ionic liquid and Si-Sal-SSIL. This was done by adding 0.25 g of extractant to 12 mL of 200 mg L^−1^ Pb(ii) nitrate solution in polypropylene bottles. The bottles were put into an orbital shaker and shaken at 400 rpm for 30 minutes at room temperature. Afterwards, the mixtures were transferred to centrifuge tubes and centrifuged at 4000 rpm for 15 minutes to separate the solid extractant and the aqueous solution into two distinct layers. The supernatant was then extracted and the final concentration of Pb(ii) ions were analysed using atomic absorption spectroscopy (AAS). This procedure was duplicated for subsequent removal studies and with 12 mL of 200 mg L^−1^ Ni(ii) nitrate solution. The percentage removal efficiency (RE, %) and the removal capacity (*q*_e_) were calculated using eqn (1) and (2), respectively.^35^1
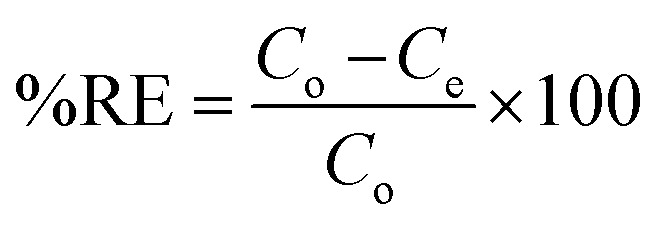
2
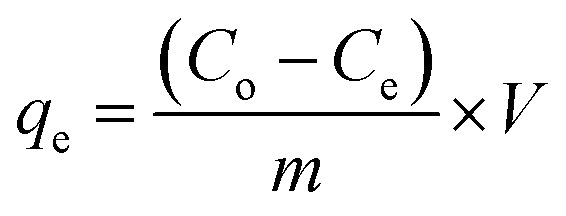
where *q*_e_ (removal capacity) represents the amount of metal ions removed at equilibrium (mg g^−1^), *C*_o_ and *C*_e_ (mg L^−1^) are the initial and equilibrium concentrations of metal ions in the aqueous solution while *V* (L) and *m* (g) represent the volume of the metal aqueous solution and mass of the extractant, respectively.

Batch adsorption studies were performed in the laboratory by varying the parameters that affects the adsorption of Pb(ii) and Ni(ii) using Si-Sal-SSIL extractant. The effect of loading ratios of activated silica gel to ionic liquid, pH, mixing time and initial concentration were studied by taking 12 mL of Pb(ii) and Ni(ii) ions aqueous solution in separate propylene bottles at room temperature. Different loading ratios of activated silica gel to ionic liquid *i.e.*, 1 : 0.5, 1 : 0.2, 1 : 0.12 and 1 : 0.1 were used while all other parameters were kept constant. pH was varied from 3 to 9 which was adjusted using 0.1 M HCl and 0.1 M NaOH. The mixing time was changed from 2 to 180 minutes. After the predetermined time was achieved, the adsorbate solution was withdrawn by centrifugation to separate the solid extractant. The final concentration of the solution was determined using AAS.

### 2.6 Adsorption kinetics

Adsorption kinetics was performed using 12 mL of 200 mg L^−1^ metal ions solution containing 0.25 g of Si-Sal-SSIL extractant with stirring at 400 rpm. The mixture was collected at a designated time interval (2, 5, 10, 15, 30, 45, 60, 80, 100, 120, 140, 160 and 180 min). The removal efficiency and removal capacity for each mixing time was calculated.

The kinetic models were then used to evaluate the Si-Sal-SSIL's performance, the mechanism involved in the competitive mass transfer of targeted analytes to the adsorbent, and the adsorption rate. Therefore, in this study, the models of pseudo first order (PFO) and pseudo second order (PSO) were employed for evaluation.^36^ Both equations were expressed in eqn (3) and (4), respectively.^37^3
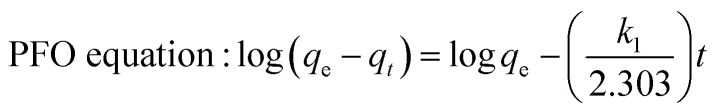
4
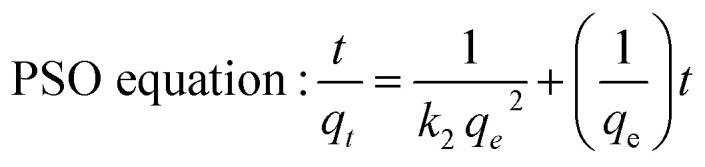
where *q*_e_ (mg g^−1^) is the amount of analytes adsorbed at equilibrium, *q*_*t*_ is the amount of analytes adsorbed at a designated time (*t*), *t* is time (min), *k*_1_ is the rate constant of pseudo first order (min^−1^) and *k*_2_ is the rate constant of pseudo second order (g mg^−1^ min^−1^).

### 2.7 Adsorption isotherm

The adsorption isotherm studies were carried out to evaluate the relationship between the equilibrium adsorbate concentration in the liquid phase and the adsorption amount on the solid phase at a room temperature. The initial concentrations of metal ions solution used for the isotherm study were 10, 30, 50, 100, 150 and 200 mg L^−1^. The equilibrium removal capacity of Si-Sal-SSIL extractant (*q*_e_, mg g^−1^) for Pb(ii) and Ni(ii) ions were calculated based on eqn (2).

Isotherm models (Langmuir and Freundlich) were used to evaluate the properties of adsorbents, adsorption mechanism, and maximum adsorption capacity. Langmuir's equation model suggests that the adsorption mechanism will occur at a specific homogenous site of the adsorbent. Langmuir's model equation is shown as follows:5
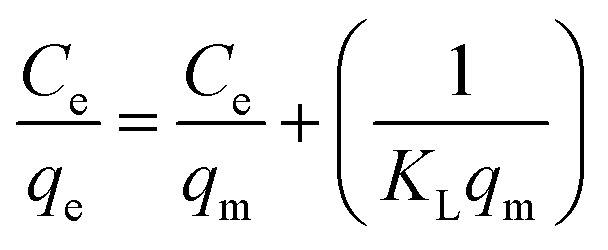
where *C*_e_ is the concentration of adsorbate at equilibrium (mg L^−1^), *q*_e_ is the amount of analytes adsorbed at equilibrium (mg g^−1^) and *q*_m_ is the maximum quantity of adsorbate adsorbed (mg g^−1^).

Freundlich's model equation describes the adsorption mechanism occurring on heterogeneous surfaces with various adsorption sites and energy levels of adsorption. Freundlich's model equation:6
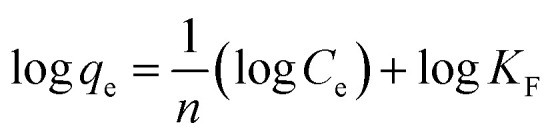
where *n* is the Freundlich constant and *K*_F_ is the adsorption capacity (mg^1−1/*n*^ L^1/*n*^ g^−1^).

## 3 Results and discussion

### 3.1 Characterization of [MTMSPI][Sal] ionic liquid and Si-Sal-SSIL extractant

**Fig. 3:**
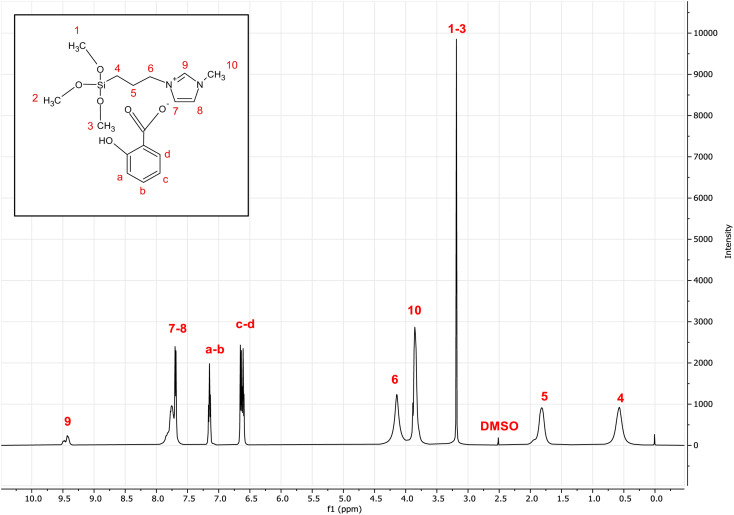
^1^H-NMR spectra of the newly synthesized 1-methyl-3-(3-trimethoxysilylpropyl) imidazolium salicylate ([MTMSPI][Sal]) ionic liquid.

Fig. 3 shows the ^1^H NMR spectrum of the [MTMSPI][Sal] ionic liquid. The NMR spectrum for the salicylate anion can be summarised as follows: *δ* 6.89 (2H, CH), *δ* 7.13 (1H, CH), *δ* 7.6 (1H, CH). As for the 1-methyl-3-(3-trimethoxysilylpropyl) imidazolium cation: *δ* 0.55 ppm (2H, CH_2_), *δ* 1.77 ppm (2H, CH_2_), *δ* 3.37 ppm (9H, CH_3_), *δ* 3.84 ppm (3H, CH), *δ* 4.13 ppm (2H, CH_2_), *δ* 7.74 ppm (2H, CH), *δ* 9.4 ppm (1H, CH). The chemical shifts of these peaks (*i.e.*, whether they will be downfield or upfield) is based on the degree of electron shielding of their respective protons. The degree of electron shielding the proton experiences is based on the electronegativity of the neighbouring atoms. The higher the electronegativity, the less shielding it experiences. Less shielding causes the peak to appear in the spectrum more downfield, whereas more shielding will cause the peak to appear upfield.

**Fig. 4:**
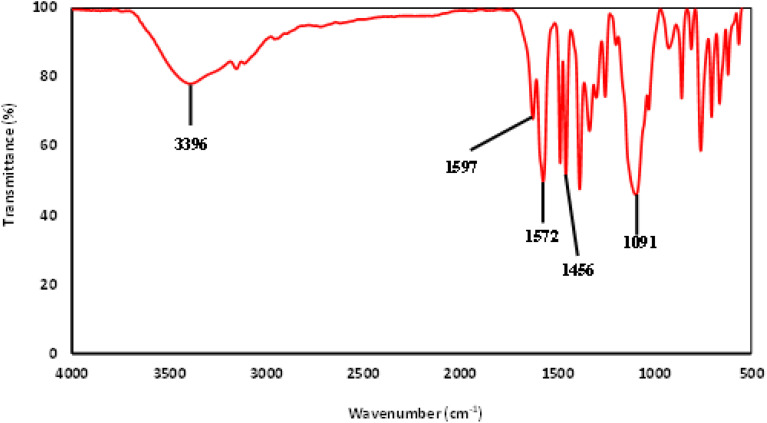
FTIR spectra of the newly synthesized 1-methyl-3-(3-trimethoxysilylpropyl) imidazolium salicylate ([MTMSPI][Sal]) ionic liquid.

The FTIR spectrum of the IL is shown in Fig. 4. A strong peak is observed at 1091 cm^−1^ from Si–O bonds in the 3-trimethoxysilylpropyl group.^38^ Next, a peak corresponding to CH_2_ scissoring from the propyl chain of the 3-trimethoxysilylpropyl group is seen at 1456 cm^−1^. The peak at 1572 cm^−1^ comes from the C–N of the imidazolium. Furthermore, the peak at 1597 cm^−1^ seems to correspond to C=O stretching from the carboxylic acid group of the salicylate anion. A broad peak is observed at 3396 cm^−1^, stemming from O–H stretching. From the FTIR and NMR spectrums, [MTMSPI][Sal] ionic liquid was confirmed to be synthesised.

The results for ion chromatography of [MTMSPI][Sal] ionic liquid is shown in Table 1. The chloride content was found to be 44.715 ppm, corresponding to [MTMSPI][Sal] with purity of 99.44%. This indicates that the synthesised [MTMSPI][Sal] is pure with a low chloride content and that the NaCl by-product formed when synthesizing the [MTMSPI][Sal] has been removed well. The water content of the [MTMSPI][Sal] was analysed using Karl Fischer titration by coulometric method. The average water content of the three replicates was measured to be 0.1527%, indicating a low water content of the synthesised [MTMSPI][Sal] ionic liquid.

**Table 1:** Chloride content and the purity of the synthesized [MTMSPI][Sal] ionic liquid

Chloride content (ppm)	Concentration (%)	Purity (%)
44.72	0.56	99.44

Elemental composition analysis of the salicylate-based ionic liquid was carried out using CHN technique. This study was done to determine the percentages of nitrogen (N), carbon (C), and hydrogen (H) in the synthesized ionic liquid and compared the values with the theoretical percentages as shown in Table 2. Percentages of 48.92%, 6.28% and 6.68% for carbon (C), hydrogen (H) and nitrogen (N), respectively, confirmed the formation of [MTMSPI][Sal] ionic liquid.

**Table 2:** Percentages of carbon (C), hydrogen (H) and nitrogen (N) in the [MTMSPI][Sal] ionic liquid

Element	Theoretical (%)	Experimental (%)
Carbon	53.38	48.92
Hydrogen	6.87	6.28
Nitrogen	7.33	6.68

**Fig. 5:**
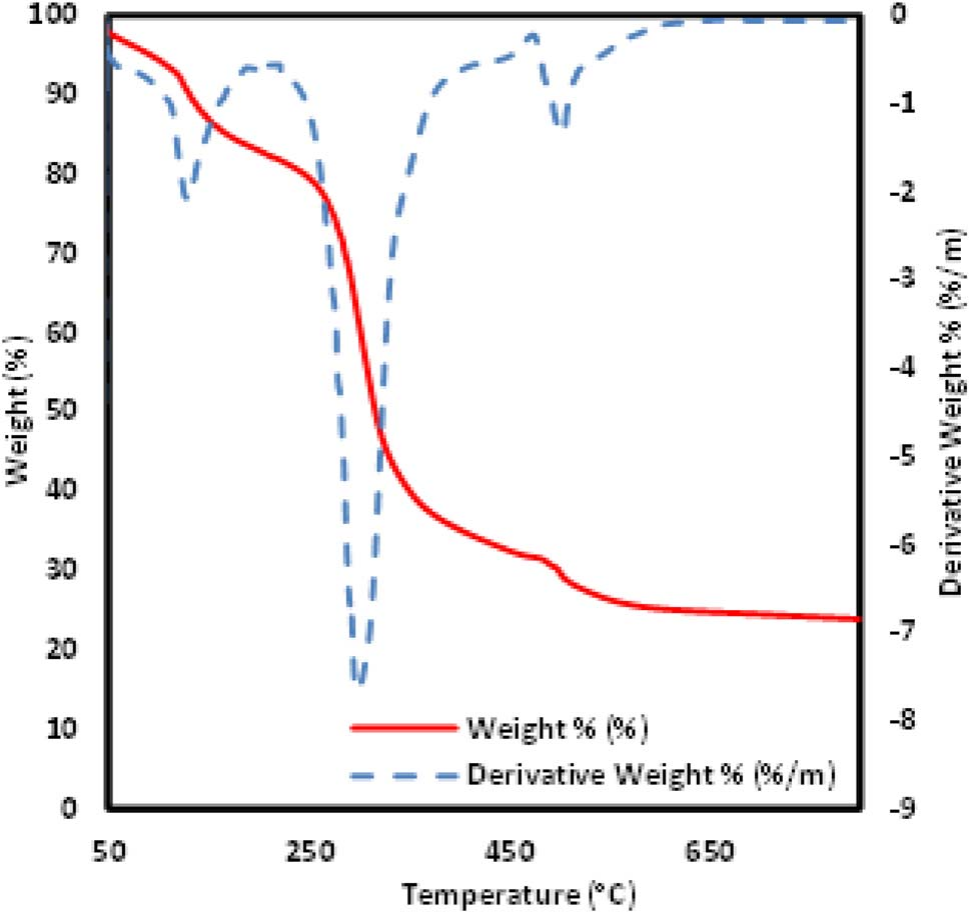
TGA and DTG curve of [MTMSPI][Sal] ionic liquid.

The thermal stability of the novel ionic liquid was studied in the range of 50 °C to 800 °C by using thermogravimetric analyzer (TGA). As shown in Fig. 5, three characteristic decomposition stages were observed. The first TGA curve shows a mass loss of about 12% up to 129 °C due to removal of adsorbed water molecule.^39^ The second weight loss (78%) occurred from 234 °C to 422 °C which was assigned to the degradation of salicylate group.^40^ Further weight loss (31%) was noticed from 490.87 °C could be associated with the degradation of remaining organic molecules.^41^ Meanwhile, as can be seen from DTG results, the maximum degradation temperature of [MTMSPI][Sal] ionic liquid was occurred at 299.08 °C.

Table 3 shows the results from the BET analysis of activated silica gel and Si-Sal-SSIL before and after washing with the solvent (dichloromethane). When comparing the surface area of the pure activated silica gel to the Si-Sal-SSIL, the activated silica gel has a much higher surface area and larger pore diameter than both SSIL samples. This is expected, as the activated silica gel does not contain any [MTMSPI][Sal] to cover the surface and fill pores. However, the Si-Sal-SSIL extractant shows a higher surface area after washing of 207.60 m^2^ g^−1^ compared to 180.4213 m^2^ g^−1^ before washing, indicating that the weakly bonded physisorbed [MTMSPI][Sal] has been washed away. This is important because physisorbed IL will contribute to leaching if not washed. The pore volume and diameter of the SSIL, both before and after washing are significantly lower than the pure silica. This result may be due to the pores of the silica being filled with [MTMSPI][Sal], which reduced the pore volume and diameter of the resulting SSIL.

**Table 3:** BET analyses of activated silica gel, Si-Sal-SSIL before and after washing

Extractant	BET surface area (m^2^ g^−1^)	Pore volume (cm^3^ g^−1^)	Pore diameter (nm)
Activated silica gel	275.81	0.70	11.52
Si-Sal-SSIL (before washing)	180.42	0.43	6.82
Si-Sal-SSIL (after washing)	207.60	0.49	6.91

**Fig. 6:**
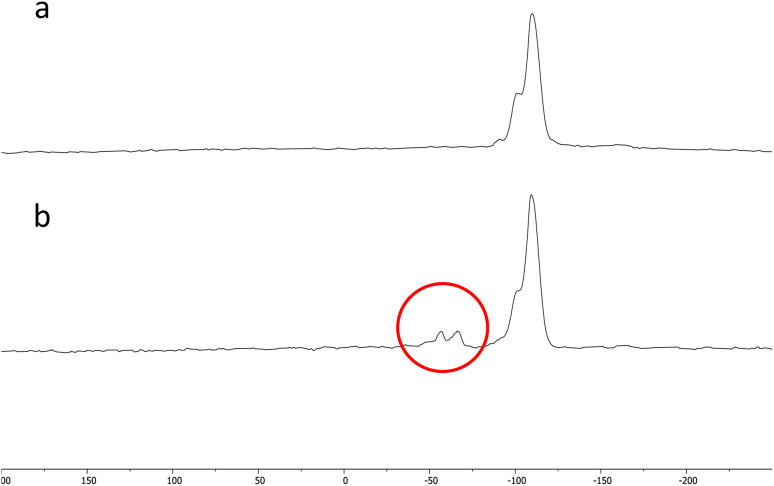
^29^Si NMR spectra of (a) pure silica and (b) Si-Sal-SSIL (modified activated silica gel with [MTMSPI][Sal] ionic liquid).

The results of the solid-state NMR analysis are shown in Fig. 6. Two small peaks are present at −57 and −66 ppm in the NMR analysis of the Si-Sal-SSIL that are not present in the analysis of the pure activated silica gel. These two peaks correspond to (SiO)_2_SiR and (SiO)_3_SiR, respectively.^42^ The presence of the two peaks confirms that the [MTMSPI][Sal] ionic liquid is covalently bonded to the activated silica gel through Si–O covalent bonds.^42^

### 3.2 Removal study

#### 3.2.1 Control study

Table 4 shows the results of the control study for the Pb and Ni metal removal. The Si-Sal-SSIL extractant has significantly higher removal capacities than the activated silica gel. This is expected due to the presence of [MTMSPI][Sal] ionic liquid in the Si-Sal-SSIL. However, as expected, the Si-Sal-SSIL has slightly lower removal capacities compared to the [MTMSPI][Sal]. This could be due to the greater capability of [MTMSPI][Sal] to remove metal ions as compared to SSIL which has less chemically immobilized [MTMSPI][Sal].

**Table 4:** Removal capacities of activated silica gel, [MTMSPI][Sal] and Si-Sal-SSIL control samples to remove Pb(ii) and Ni(ii) ions from aqueous solution

Extractant	Removal capacity (mg g^−1^)	
	Pb	Ni
Activated silica gel	1.37	2.35
[MTMSPI][Sal]	5.57	5.01
Si-Sal-SSIL	5.37	4.60

#### 3.2.2 Effect of loading ratios of activated silica gel to ionic liquid

**Fig. 7:**
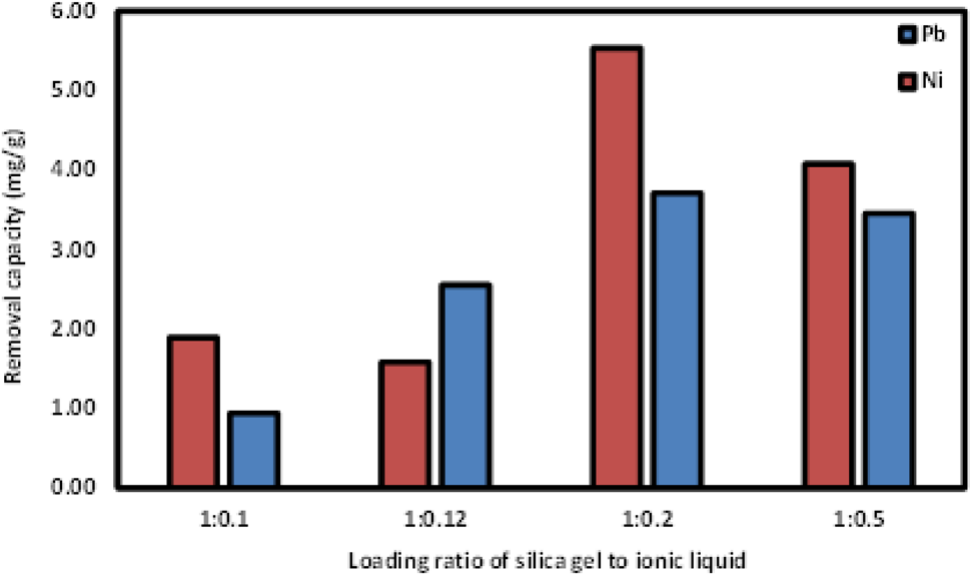
The effect of different loading ratios of activated silica gel : [MTMSPI][Sal] on the removal process of Pb(ii) and Ni(ii) ions onto Si-Sal-SSIL.

Fig. 7 compares the removal capacities of Si-Sal-SSIL with different loading ratios of activated silica gel to [MTMSPI][Sal] in the removal of Pb(ii) and Ni(ii) ions. Loading ratio of 1 : 0.2 showed the highest removal capacity, followed by 1 : 0.5. The lowest removal capacity was from Si-Sal-SSIL with loading ratio of 1 : 0.1, likely due to the low amount of [MTMSPI][Sal] contained. This trend continued in the removal of Ni(ii) as shown in Fig. 7. Further studies were proceeded using Si-Sal-SSIL with loading ratio of 1 : 0.2, due to the optimum loading ratio and achieved highest removal capacity for both metal ions.

#### 3.2.3 Effect of pH

**Fig. 8:**
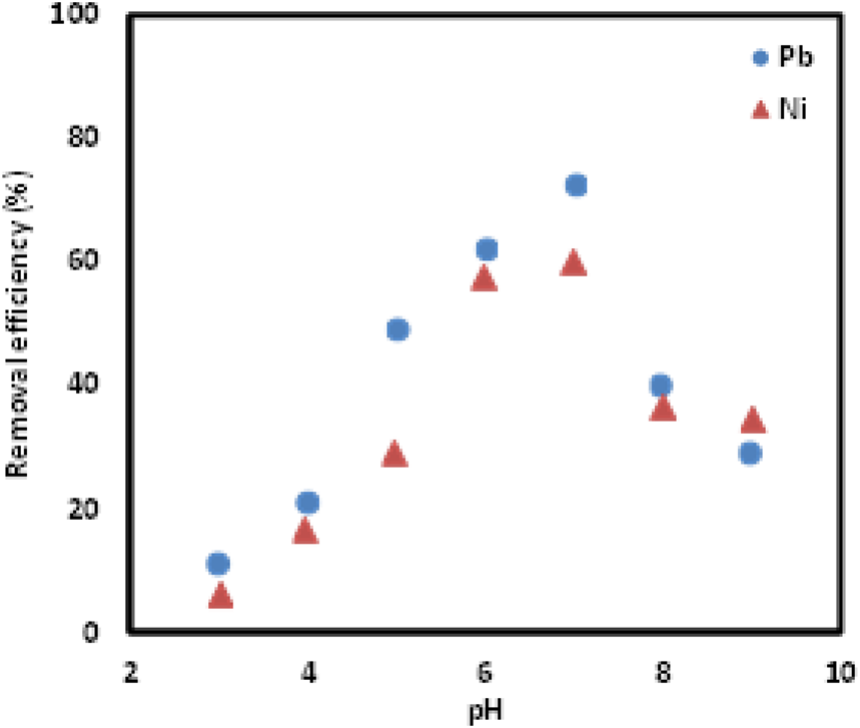
Effect of pH on the removal of Pb(ii) and Ni(ii) ions from aqueous solution using Si-Sal-SSIL.

Initial pH of the adsorption system has significant role in adsorption of sorbate, as it affects the surface morphology of the sorbent and binding nature of sorbate, hence, affecting the removal percentages. Therefore, the effect of sample pH was investigated at pH ranging from 3 to 9. The findings showed a significant effect of sample pH on the removal of Pb(ii) and Ni(ii) ions. Based on the findings presented in Fig. 8, the highest removal percentage was observed at pH 7, where the compounds existed in protonated forms. In alkaline condition, the removal efficiency of Si-Sal-SSIL extractant for both metal ions decreased as precipitation of metal hydroxide formed. In acidic condition, the amount of metal ions removed from the aqueous solution lower compared to the neutral state, which is due to the existence of protons provided by other functional groups that contain lone pairs.^43^

#### 3.2.4 Effect of mixing time

**Fig. 9:**
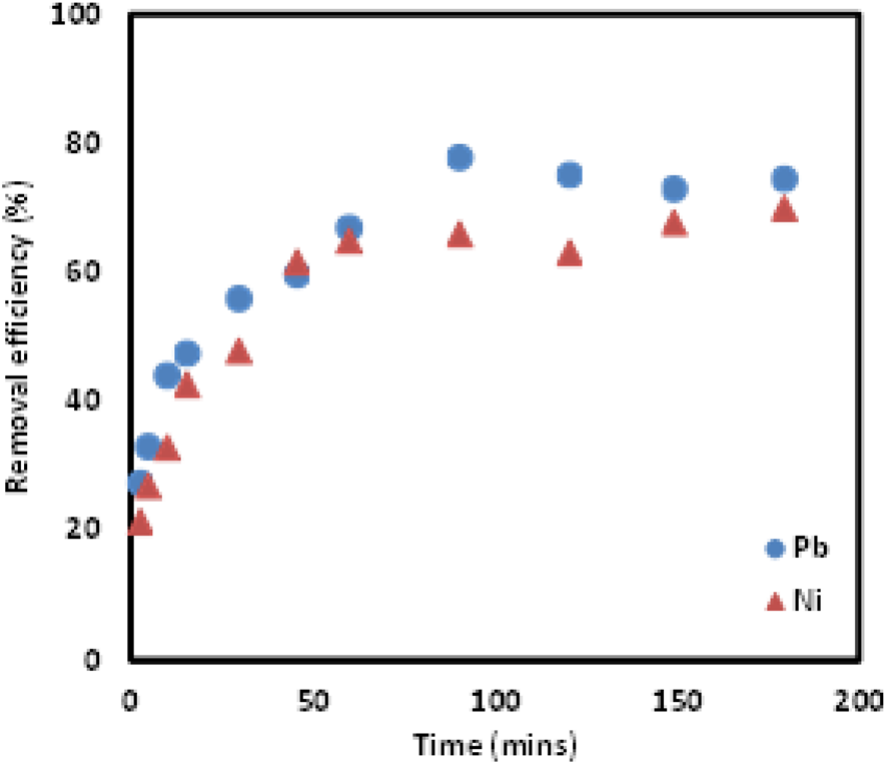
Effect of mixing time on Pb(ii) and Ni(ii) ions removal efficiencies using Si-Sal-SSIL.

The effect of mixing time on the adsorption of Pb(ii) and Ni(ii) ions onto Si-Sal-SSIL extractant was evaluated and presented in Fig. 9. The mixing time varied from 2 to 180 minutes under neutral conditions with constant amount of extractant (0.25 g), initial concentration (200 mg L^−1^), and shaking speed (400 rpm). Maximum adsorption was achieved at 100 minutes time interval and no significant increase found by further increase in time. Initially, the rapid removal of both metal ions was due to the availability of vacant places on the surface of adsorbent, which caused the metals to interact with the adsorbent and hence, there was continuous increase in adsorption capacity by increasing time slot from zero to 100 minutes. However, the removal capacity was reduced as the contact time increased until the equilibrium is achieved.

#### 3.2.5 Effect of initial concentration

**Fig. 10:**
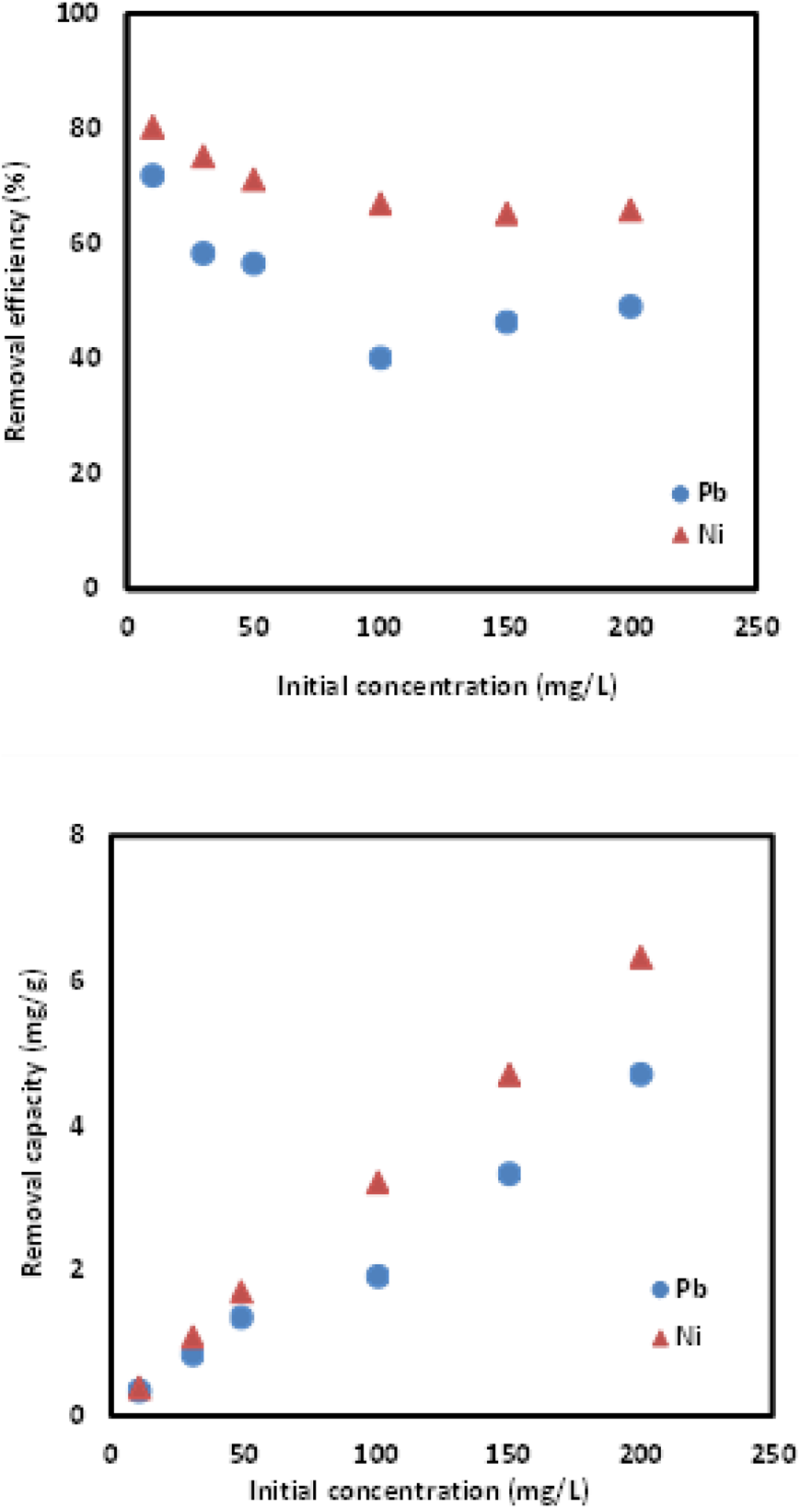
Effect of initial concentration on Pb and Ni metal removal efficiencies and removal capacities of Si-Sal-SSIL.

Initial concentration of adsorbate can affect the adsorption phenomenon. For this purpose, initial concentration of Pb(ii) and Ni(ii) ions solution was varied in range of 10–200 mg L^−1^, while the extractant was kept constant at 0.25 g. Results in Fig. 10 indicate that the highest removal capacities obtained were at 200 mg L^−1^ for both metal ions with removal capacities of 4.7059 mg g^−1^ and 6.3197 mg g^−1^, respectively. The higher the initial concentration, the higher removal capacities achieved by Si-Sal-SSIL in extracting Pb(ii) ions from aqueous solution due to the vacant spaces available on the surface of adsorbent.^44^ However, it can be seen that the removal percentages decreased with increasing initial concentration of metal ions solution due to the competition for adsorption sites among the metal ions.

#### 3.2.6 Reusability study

**Fig. 11:**
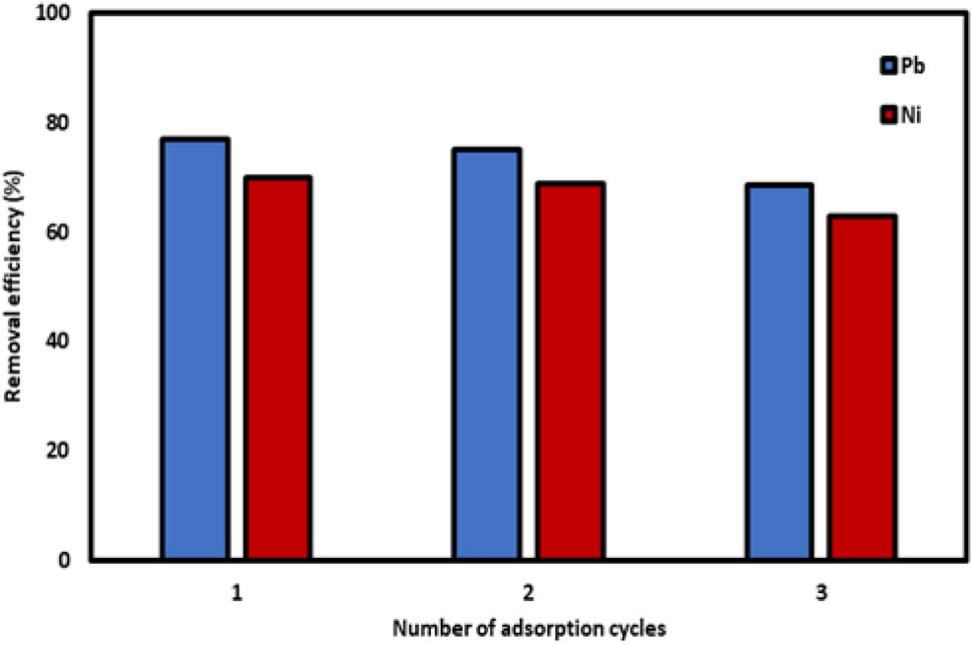
Reusability test of Si-Sal-SSIL extractant in removing Pb(ii) and Ni(ii) ions from aqueous solution.

The reusability of Si-Sal-SSIL adsorbent in removing Pb(ii) and Ni(ii) metal ions was evaluated through three identical sorption–desorption cycles. The results, depicted in Fig. 11, showed that the removal efficiencies of Si-Sal-SSIL on the first and second cycles decreased slightly by 1–2% for Pb(ii) and Ni(ii) ions. Meanwhile, it can be seen in the third cycle, both metal ions showed the same trend, whereby the removal efficiencies were dropped about 6%. The largest decrease in reusability efficiency occurred after the second cycle for both metal ions. Overall, the novel Si-Sal-SSIL adsorbent could be reused for up to three cycles with minimal loss of efficiency, demonstrating its potential for cost-effective metal ion extraction from aqueous solution.

### 3.3 Evaluation of the adsorption data through kinetic fitting models

**Fig. 12:**
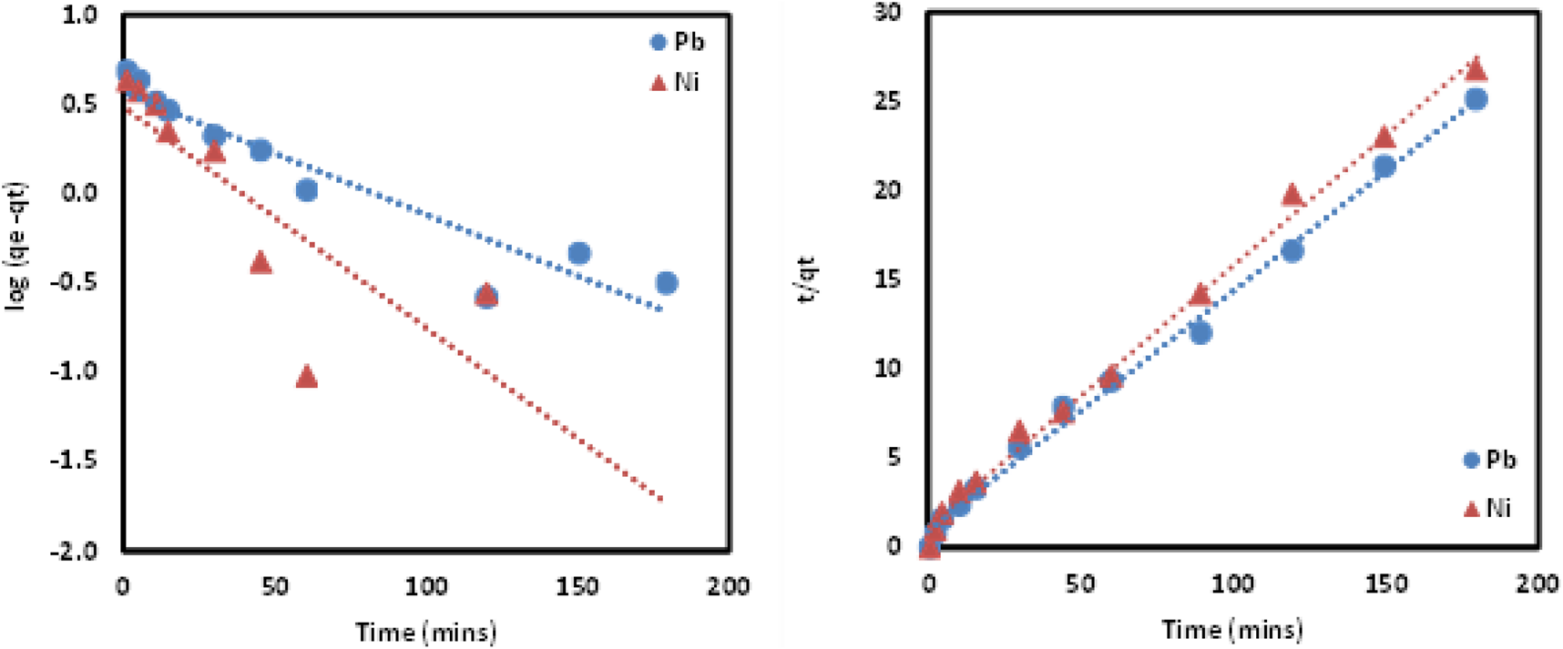
PFO and PSO kinetic models of the removal of Pb(ii) and Ni(ii) from aqueous solution by Si-Sal-SSIL.

Adsorption kinetics is crucial in determining Pb and Ni metal ions uptake rate and time needed for the metal adsorption process. In this work, kinetic study was conducted at different time intervals for Pb and Ni adsorption by applying the linearized form of pseudo first order (PFO) and pseudo second order (PSO) kinetic model as shown in eqn (3) and (4). For PFO kinetic model, log(*q*_e_ − *q*_*t*_) was plotted against time to get the values of *k* and *q*_e_ from the slope and intercept of the graph, respectively. Meanwhile, *t*/*q*_*t*_*vs. t* was graphed to find the PSO kinetic model. Based on the results presented in Table 5 and Fig. 12, coefficient of determination (*R*^2^) values for PSO was higher as compared to the PFO, for both metal ions. This suggesting that the adsorption process for Pb and Ni ions onto Si-Sal-SSIL extractant was dominated by chemical adsorption process. This may be due to the affinity of the salicylate functional group to metal ions.^33^

**Table 5:** Kinetics model for the removal of Pb and Ni ions by the Si-Sal-SSIL extractant

Pseudo First Order			Pseudo Second Order		
	Pb	Ni		Pb	Ni
*q*_e exp_ (mg g^−1^)	7.4736	6.3298	*q*_e exp_ (mg g^−1^)	7.4736	6.3298
*q*_e cal_ (mg g^−1^)	1.7692	1.6247	*q*_e cal_ (mg g^−1^)	7.4294	6.306
*k*_1_ (min^−1^)	0.0030	0.0054	*k*_2_ (g mg^−1^ min^−1^)	0.0200	0.0185
*R* ^2^	0.9015	0.6358	*R* ^2^	0.9952	0.9948

Aside from that, the calculated adsorption capacities (*q*_e cal_) for PSO model were found to be very close to the experimental values. Furthermore, the rate constant (*k*_2_) for PSO was quite high as opposed to PFO, indicating a faster adsorption rate for both metal ions onto Si-Sal-SSIL extractant. A higher adsorption rate will allow Pb and Ni metal ions to bind with the active site of SSIL faster than the remaining analytes. They will fight for the few active sites left if the adsorption rate is low, resulting in low *q*_e_ values.^45^ Hence, PSO kinetic model was found to be the fittest model to explain the sorption of Pb and Ni metal ions.

### 3.4 Evaluation of the adsorption data through isotherm fitting models

**Fig. 13:**
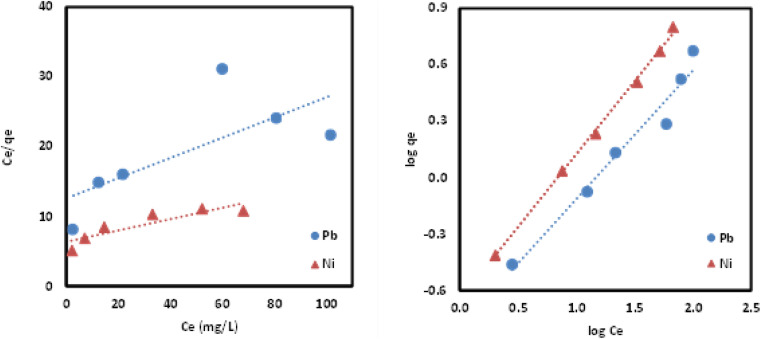
Linear plots of Langmuir and Freundlich isotherm model for the adsorption of Pb(ii) and Ni(ii) onto Si-Sal-SSIL extractant.

The equilibrium data for uptake of Pb(ii) and Ni(ii) metal ions onto Si-Sal-SSIL were further evaluated by adsorption isotherm models, namely, Langmuir and Freundlich models. Langmuir isotherm was developed for the homogeneous system, and it gives concept of monolayer adsorption of solute on the surface of sorbent.^44^ Distribution of metal ions between liquid and solid surface was calculated by eqn (5) and (6). The important feature of Langmuir is commonly presented in terms of dimensionless separation factor, *R*_L_. The value of *R*_L_ was computed using the following equation:^46^7
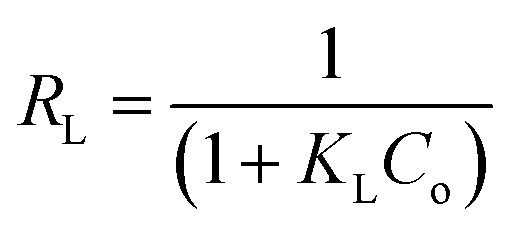
where *K*_L_ is the Langmuir constant (L mg^−1^) and *C*_o_ represented the initial concentration of the solution (mg L^−1^), The values of *R*_L_ obtained for Pb(ii) and Ni(ii) ions were 0.8969 and 0.8865, respectively. This indicated that the adsorption of Pb(ii) and Ni(ii) metal ions onto Si-Sal-SSIL extractant is a favourable adsorption. However, low *R*^2^ values in the Langmuir model reflected a poor fitting of the model to the adsorption data of both metal ions. Freundlich isotherm plots (log *q*_e_*vs.* log *C*_e_) were displayed in Fig. 13 and isotherm parameters were reported in Table 6. The Freundlich model proved to be promising in explaining the multilayer adsorption of both metal ions due to the high *R*^2^ values (0.97–0.99) and nearly perfect fitting using both regression forms compared to Langmuir model. The fitting of Freundlich model by the Si-Sal-SSIL indicated the heterogeneous nature, where they interacted with the Pb(ii) and Ni(ii) metal ions *via* multilayer coverage. In addition, the suitability of the model was further confirmed by the dimensionless factor (*n*), which was greater than 1 for both metal ions, implying the surface heterogeneity of Si-Sal-SSIL and better adsorption performance.^43^

**Table 6:** Parameters of Langmuir and Freundlich isotherms for the removal of Pb(ii) and Ni(ii) ions by Si-Sal-SSIL extractant

Langmuir			Freundlich		
	Pb	Ni		Pb	Ni
*q*_m_ (L mg^−1^)	6.9061	12.3153	*K*_F_ (mg g^−1^)(L mg^−1^)1/*n*	0.4507	0.5226
*K*_L_ (min^−1^)	0.0115	0.0128	*n*	1.4641	1.2917
*R* ^2^	0.5429	0.8008	*R* ^2^	0.9655	0.9984

### 3.5 Adsorption mechanism

The mechanism of adsorption using Si-Sal-SSIL involves the hydroxyl group and oxygen of the salicylate functional group in the extractant, which act as electron donors to facilitate the chemisorption of Pb(ii) and Ni(ii) ions. Furthermore, the presence of both organic and inorganic constituents in the Si-Sal-SSIL extractant creates more active sites for the binding of both metal ions in the aqueous solution. Additionally, Pb(ii) and Ni(ii) ions may be trapped within the pores of Si-Sal-SSIL, contributing to the overall removal efficiency. Therefore, the enhanced removal efficiency of metal ions by Si-Sal-SSIL is likely due to the presence of thiol functional groups on the extractant surface, which can bind with the ions.

## 4 Conclusions

In summary, [MTMSPI][Sal] was synthesized and characterized using ^1^H-NMR and FTIR to ascertain the structure. Through the characterization, [MTMSPI][Sal] was confirmed to be synthesized. In addition, ion chromatography, CHN and Karl-Fischer titration was done to measure the purity of the prepared [MTMSPI][Sal] and it was found to have both low chloride content and low water content. The [MTMSPI][Sal] was then chemically immobilized onto activated silica gel to produce Si-Sal-SSIL extractant. BET analysis was then performed to compare the surface area of the prepared Si-Sal-SSIL before and after washing. It was found that the surface area after washing was lower, indicating that weakly physisorbed IL has been removed and leaching is less likely to occur. Furthermore, solid-state NMR confirmed the presence of covalent bonds between the [MTMSPI][Sal] and silica support. From the various removal studies, it was found that the optimum loading ratio of activated silica gel to [MTMSPI][Sal] was 1 : 0.2 for the removal of Pb(ii) and Ni(ii). Additionally, the optimum pH and mixing time was determined to be 7 and 100 minutes, respectively to ensure maximum removal of both Pb(ii) and Ni(ii). Kinetic adsorption modelling was carried out and the pseudo-second order (PSO) model was determined to be a better fit for both metal ions. Isotherm study was conducted and the Freundlich isotherm was found to be the model that fit the removal study data best. A comparison of the performance of the Si-Sal-SSIL in the removal of these two metal ions showed that Pb(ii) and Ni(ii) ions had comparable affinity to the Si-Sal-SSIL at high concentrations but lower affinity at lower concentrations. From this study, SSIL containing salicylate functional group was shown to be a promising removal agent for the removal of Pb(ii) and Ni(ii) ions from single aqueous solution.

## Author contributions

Nur Anis Liyana Kamaruddin and Avisenna Muhammad Romil: methodology, investigation; Nur Anis Liyana Kamaruddin: writing-the original draft; Nur Anis Liyana Kamaruddin and Avisenna Muhammad Romil: formal analysis; Mohd Faisal Taha: validation; Fairuz Liyana Mohd Rasdi: reviewing and editing; Mohd Faisal Taha: conceptualization, supervision, funding acquisition.

## Conflicts of interest

There are no conflicts to declare.

## Supplementary Material
